# Microreactor Array Device

**DOI:** 10.1038/srep08736

**Published:** 2015-03-04

**Authors:** Peter Wiktor, Al Brunner, Peter Kahn, Ji Qiu, Mitch Magee, Xiaofang Bian, Kailash Karthikeyan, Joshua LaBaer

**Affiliations:** 1 Engineering Arts LLC, Tempe, Arizona, U.S.A; 2 The Virginia G. Piper Center for Personalized Diagnostics, Biodesign Institute, Arizona State University, Tempe, Arizona, U.S.A

## Abstract

We report a device to fill an array of small chemical reaction chambers (microreactors) with reagent and then seal them using pressurized viscous liquid acting through a flexible membrane. The device enables multiple, independent chemical reactions involving free floating intermediate molecules without interference from neighboring reactions or external environments. The device is validated by protein expressed *in situ* directly from DNA in a microarray of ~10,000 spots with no diffusion during three hours incubation. Using the device to probe for an autoantibody cancer biomarker in blood serum sample gave five times higher signal to background ratio compared to standard protein microarray expressed on a flat microscope slide. Physical design principles to effectively fill the array of microreactors with reagent and experimental results of alternate methods for sealing the microreactors are presented.

Biology experiments in the early 20^th^ century were performed one at a time in glassware such as test tubes, petri dishes or flasks. In the middle of the century immunoassays, based on 96-well plastic microtiter plates, were prototypical parallel biology experiments^1^. Currently around a million experiments are performed simultaneously for gene expression analysis^2^ and around a billion for next generation DNA sequencing^3^. These high throughput experiments are based on molecules tethered to a surface. However chemical reactions in living cells involve untethered, free floating molecules in aqueous solutions. Many different biochemical reactions occur simultaneously depending on cell type, cell cycle or external stimuli. Unravelling this complexity and its effect on human health requires high throughput experimental platforms that can simultaneously study thousands of biochemical reactions involving untethered, free floating, molecular compounds.

Protein expression in living cells involves untethered intermediate molecules such as mRNA, enzymes, ribosomes, amino acids and polypeptides. Proteins can also be expressed outside of living cells by subjecting gene DNA to ‘cell-free’ *in vitro* coupled transcription and translation (IVTT) reagent. This is the process used for nucleic acid programmable protein arrays (NAPPA)^4^,^5^ to express unique proteins from plasmid DNA containing their full length genes. Proteins are expressed and captured *in situ* in a microarray format at the time of assay. The microarrays are used to assay thousands of protein interactions simultaneously to discover autoantibody biomarkers correlated to specific diseases^6^,^7^,^8^,^9^,^10^,^11^,^12^ and to detect antibodies to pathogens^13^,^14^. To preserve protein function, assays using NAPPA are typically done within hours of expressing fresh proteins without ever allowing them to dry out. Contrast this with conventional protein microarrays based on purified proteins printed from frozen stock and then stored possibly for months before assay.

*In situ* protein expression for NAPPA is typically carried out on flat microscope slides by flooding the entire microarray surface with IVTT reagent. Spot to spot diffusion currently limits NAPPA density to ~2,500 protein spots per slide. Density can be increased by expressing proteins in an array of micro reaction chambers (microreactors)^15^. We report a novel device to reliably fill all of the microreactors with reagent and then completely seal them. The device is amenable to production scale processing of microreactor array slides.

## Results

### Microreactor array processing overview

**Figure 1:**
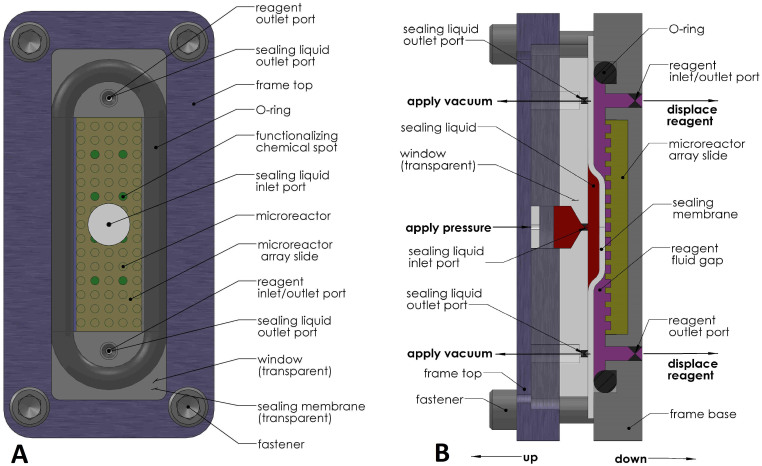
Schematic of preferred microreactor array fill & seal device. (A) Top view schematic of device consisting of a rigid frame with fasteners clamping a transparent window and transparent sealing membrane onto an O-ring sealing around the perimeter of a microreactor array slide consisting of microreactors with functionalizing chemicals. There are inlet & outlet ports for reagent at the bottom and inlet & outlet ports for sealing liquid at the top. Features are not drawn to scale. (B) Section view schematic of same device illustrating the process of displacing excess reagent and sealing microreactors. After completely filling the microreactors, excess reagent is displaced from the slide by pressurized sealing liquid injected through a sealing liquid inlet port acting on a sealing membrane. As the sealing liquid spreads out across the sealing membrane it first displaces excess reagent though reagent inlet & outlet ports and then seals the microreactors into isolated chemical reaction containers. The schematics (A, B) were drawn by Peter Wiktor.

The microreactor array platform consists of an array of functionalized microreactors in a microscope slide format and a device for filling the microreactors with reagent and then sealing them. Microreactor array slides (slides) are fabricated from silicon wafers using standard isotropic wet etch process with details provided in **Methods**. Microreactors are 270 μm across, 70 μm deep and 375 μm apart. There are ~14,000 microreactors in a hexagonal array pattern on a single 25.4 mm × 76.2 mm microscope slide format. The silicon surface is oxidized with 95 nanometer silicon dioxide (SiO_2_) which is the main component of glass. This makes the silicon slide compatible with conventional surface chemistry for functionalizing glass. It also prevents fluorescent signal quenching of bare silicon. Individual microreactors are filled with different unique functionalizing chemicals using non-contact piezoelectric inkjet dispensing technology^15^,^16^,^17^. Portions of these chemicals are bound to the functionalized surfaces of the microreactors. Dried printed slides may be stored for later processing. The slides may be soaked in a blocking buffer to wash away remaining unbound chemicals and to mitigate nonspecific binding. A centrifuge or vacuum chamber is used to force entrapped air out of the microreactors and fill them with the blocking buffer. After rinsing and drying, slides are inserted into the fill & seal device, Figure 1. An O-ring is placed around the periphery of the slide for vacuum or pressure sealing. A transparent, flexible, impenetrable, smooth, sealing membrane is placed over the O-ring and slide. A transparent window is placed over the sealing membrane and the assembly is clamped together in a rigid frame using fasteners.

Approximately 300 μl of degassed reagent is injected into a reagent inlet/outlet port and forced through a thin fluid gap between the slide and sealing membrane. Vacuum is applied to the fluid gap, through a reagent outlet port, before injecting the reagent. Microreactors are filled with reagent at a rate of greater than 10,000 microreactors per second. The functionalizing chemicals stay in place during filling since they are bound to the surface. High viscosity, pressurized, incompressible, sealing liquid is injected onto the sealing membrane through an inlet port in the window. The sealing membrane wipes excess reagent from the slide surface as the sealing liquid spreads out across the membrane. Excess reagent is forced out through inlet or outlet reagent ports and ~200 μl may be recovered. After spreading out across the whole surface, the pressurized sealing liquid continues to apply even pressure onto the slide surface through the sealing membrane. This uniformly seals all of the microreactors into isolated reaction containers.

The process of filling and sealing all of the microreactors takes less than five seconds. It is actuated entirely by opening or closing valves and is therefore amenable to automation via electronic actuators. Reagent reacts with functionalizing chemicals in the microreactors possibly creating untethered intermediate molecules that are contained for extended incubation periods. After incubation, the slide is removed from the fill & seal device for further chemical processing such as washing, blocking or labeling. To preserve molecular functionality, the slide is typically never allowed to dry out during an assay. The whole slide may be flooded with sample fluid to detect probe (query) molecules by their affinity to specific chemical products (targets) in the microreactors. After chemical processing the slide may be dried and imaged using a fluorescent microarray scanner for example.

### Alternate methods for sealing microreactors

**Figure 2:**
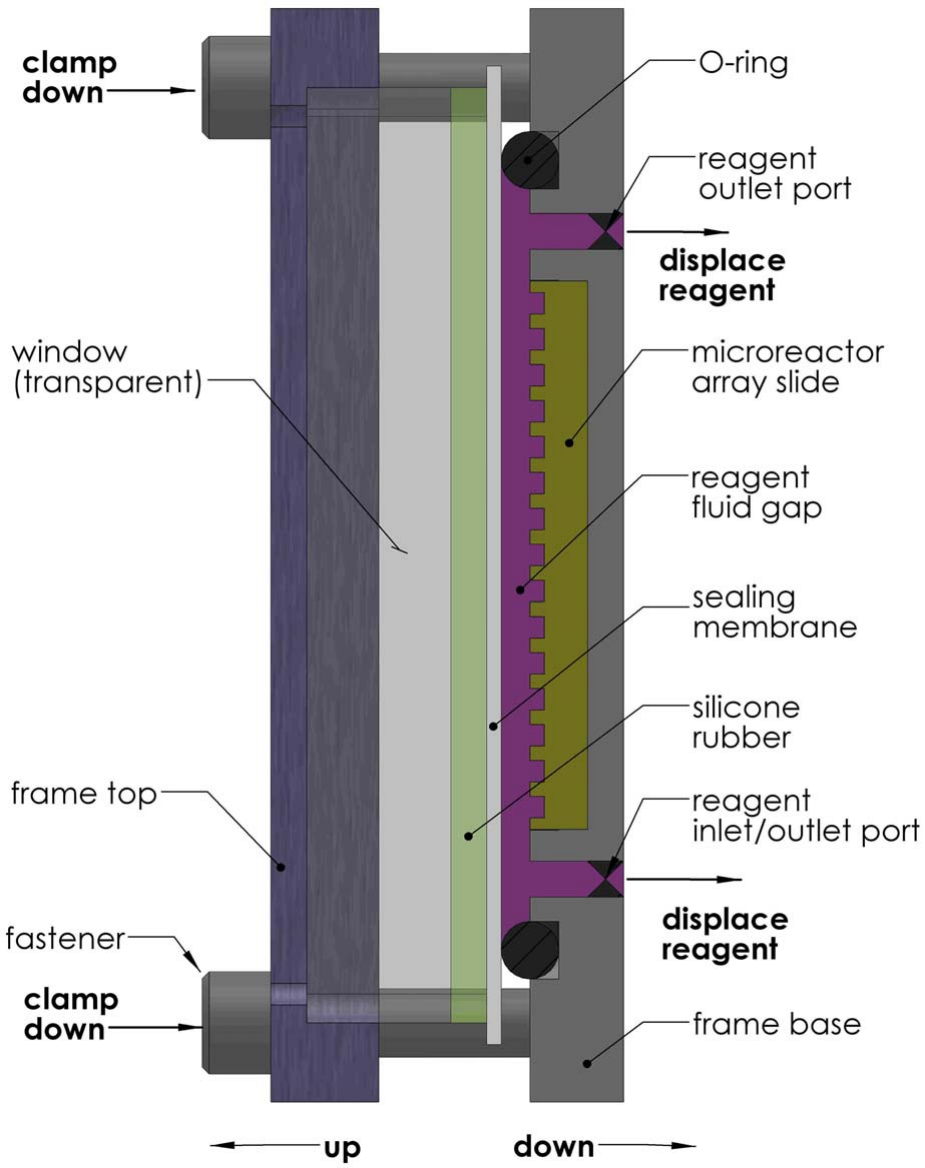
Schematic of silicone rubber based fill & seal device. Section view schematic of alternate microreactor fill & seal device based on sealing microreactors using silicone rubber instead of a viscous sealing liquid. Features are not drawn to scale. The schematic was drawn by Peter Wiktor.

Three alternate methods for sealing microreactors were evaluated. Devices were fabricated to test the alternate methods. The alternate methods use the same process of filling microreactors with reagent as the preferred device of Figure 1. All of the alternate methods also use a sealing membrane but differ in how they actuate the membrane, either by: 1) silicone rubber, 2) gas or 3) hydraulic oil. Figure 2 is a schematic representation of the device based on silicone rubber. It is similar to the device reported previously^15^ with the addition of the reagent outlet port and the sealing membrane. The reagent outlet port enables applying vacuum separately from the inlet port. Previously^15^ vacuum was applied through the reagent at the inlet port. This introduced air into the reagent which interfered with filling of the microreactors. The sealing membrane isolates the microreactors from the porous silicone rubber and thus reduces diffusion after sealing the microreactors. The other two alternate methods are similar to Figure 1 with either gas or hydraulic oil replacing the viscous sealing liquid. Pros and cons of the various approaches are summarized in **Discussion**.

### Experimental evaluation of fill & seal methods

**Figure 3:**
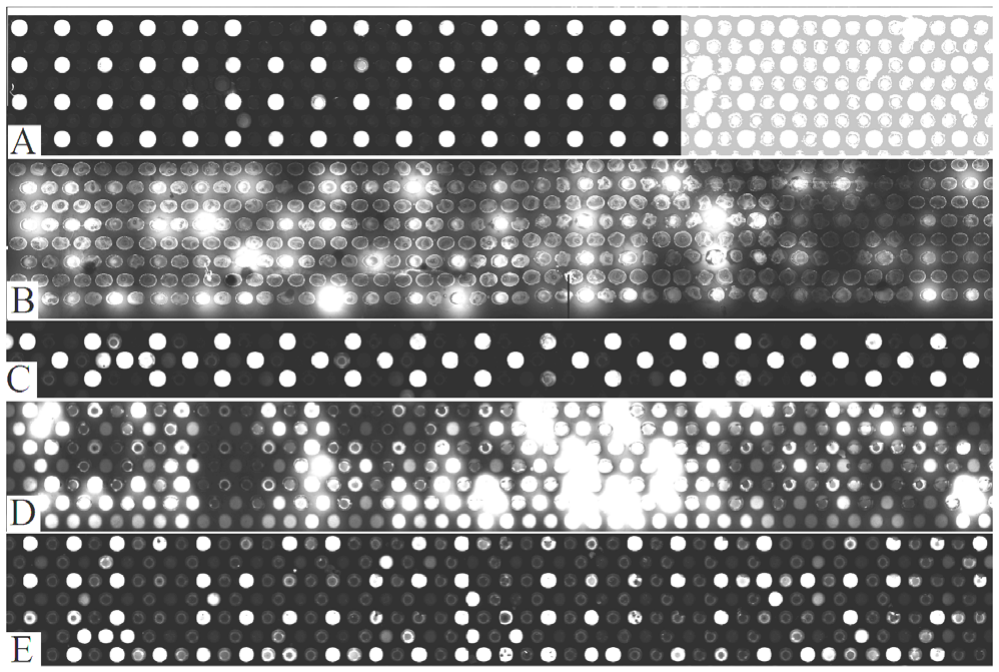
Fluorescent microarray scanner images of NAPPA subarrays for different sealing methods. Enlargements of fluorescent microarray scanner images of nucleic acid programmable protein arrays (NAPPA) subarrays processed using five different methods of sealing microreactors. Bright spots are fluorescently labeled proteins expressed *in situ* from plasmid DNA using *in vitro* coupled transcription translation (IVTT) reagent. Proteins are captured at each spot with a printing mixture containing capture antibodies. Spots adjacent to the bright spots just have the printing mixture without DNA. Therefore signal in adjacent spots indicates diffusion from the spots containing DNA. The five subarrays correspond to the following five sealing methods: (A) viscous liquid, (B) no sealing i.e flat glass, (C) silicone rubber, (D) gas, (E) hydraulic oil. Brightness and contrast of the right third of subarray (A) is separately adjusted to show actual printing density and locations of the adjacent spots. Brightness and contrast of the image as a whole is adjusted to help visualize diffusion to compare the different methods.

The alternate fill & seal methods were experimentally evaluated for NAPPA and compared to the preferred method of Figure 1 and to flat glass. Printing mixture, containing capture antibodies, is printed into microreactors followed by plasmid DNA. To clearly detect diffusion, spots containing printing-mixture & plasmid DNA are surrounded by spots containing just the printing-mixture. Any expressed proteins that diffuse away from a DNA spot are captured by the antibodies in neighboring spots and therefore easily detected. Fluorescently labeled proteins expressed from the DNA are displayed in the subarrays of Figure 3. The top subarray (A) was expressed using the preferred fill & seal device of Figure 1. There is negligible signal in the adjacent spots indicating negligible diffusion and capture of expressed proteins. This is significant since the signal at a given spot is due exclusively to the analyte at that spot and not from its neighbors. This reduces false positives during data analysis. Brightness and contrast are adjusted at the right third of the subarray in Figure 3A to show actual printing density and locations of the adjacent spots.

Standard NAPPA on flat glass is compared to NAPPA on microreactor array in Figure 3B. The same set of plasmid DNA was printed on flat glass (B) at the same time as microreactor arrays (A). Printing conditions were optimized for microreactor arrays. Genes were expressed into their corresponding proteins by flooding the whole array with IVTT reagent and then incubating for three hours. Proteins on the substrates in (A) and (B) were fluorescently labeled, scanned and displayed using identical conditions. There is more diffusion on flat glass (B) vs. microreactor array (A). However standard NAPPA typically has lower diffusion if the printing conditions are optimized for flat glass^11^. Normally, for standard NAPPA, mixtures of higher concentration DNA & printing mixture are printed using a pin-spotter at four times lower spot density. Diffusion on flat glass is much lower under these standard conditions.

**Figure 4:**
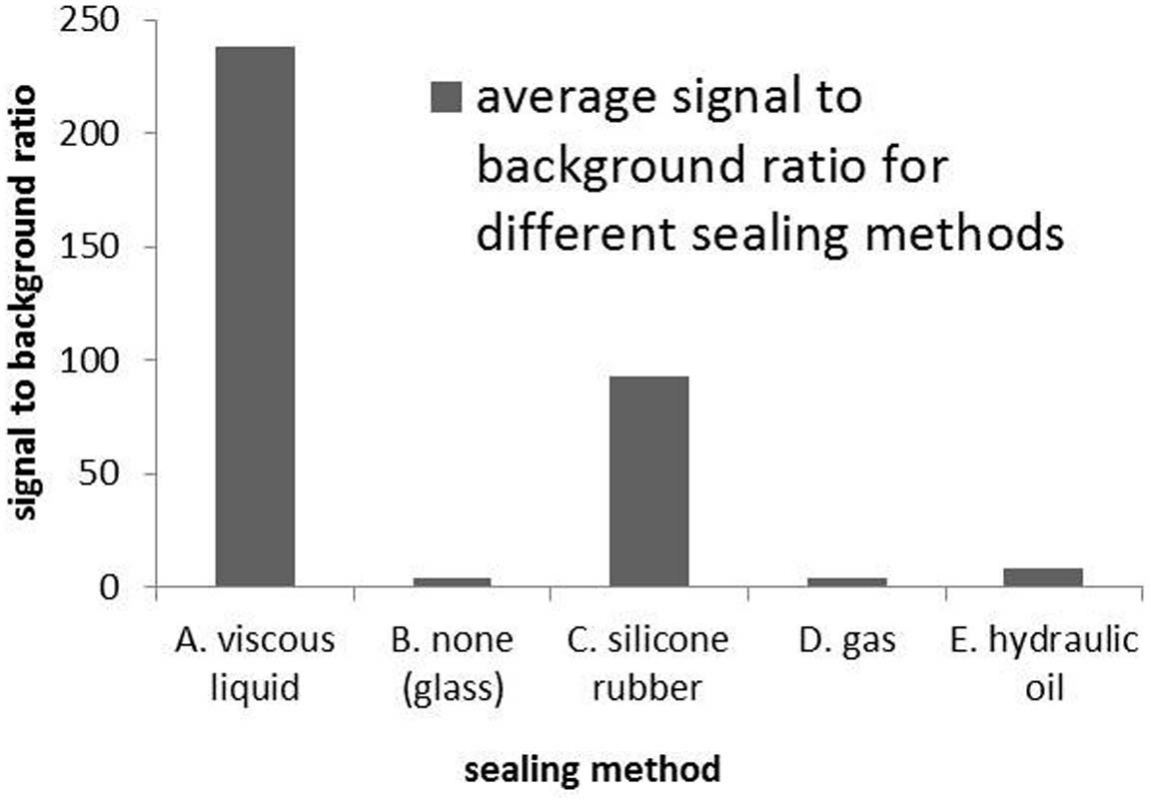
Comparison chart of microreactor sealing methods. Signal to background ratio of fluorescently labeled protein spots using five different microreactor sealing methods are shown. Signal is the average of the median pixel intensity of the protein spots from the subarrays in Figure 3. Background is the average of the median pixel intensity of the adjacent spots. Proteins were expressed *in situ* in a microarray format using NAPPA chemistry.

Protein subarrays expressed using the three alternative methods: silicone rubber, gas and hydraulic oil are shown in Figure 3 (C, D and E) respectively. Signal to background ratio of the various sealing methods are plotted in Figure 4.

### Validation of microreactor arrays as a clinical screening platform

**Figure 5:**
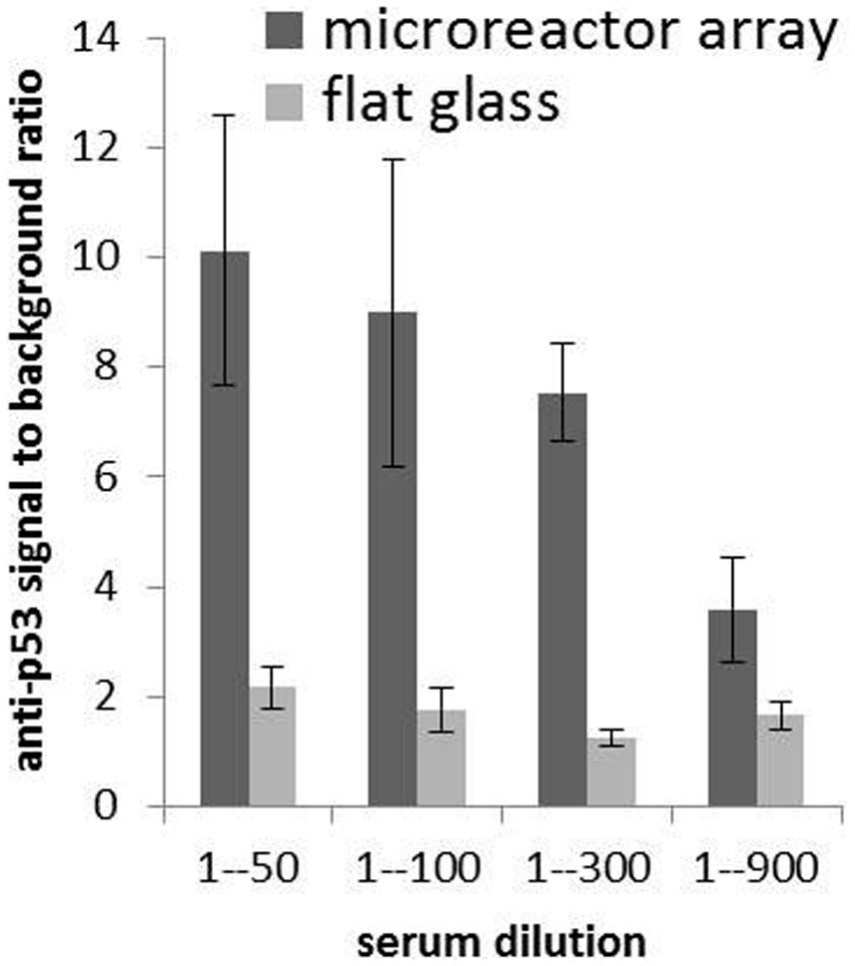
Signal to background ratio of antibody response in patient blood serum sample. Signal to background ratio of anti-p53 antibody response in cancer patient blood serum detected on NAPPA microreactor array vs. standard NAPPA on flat microscope slides. The height of the bar for each dilution point is the average signal to background ratio of six spots on the arrays. Error bars indicate signal range for the six spots. Signal of each spot is the median pixel value of the spot. Background is the median signal of all of the spots on the array.

Some cancer patients produce autoantibodies against p53, a tumor suppressor protein^18^. These autoantibodies can be detected in blood serum samples using immunoassays such as enzyme-linked immunosorbent assay (ELISA) or NAPPA. NAPPA has dynamic range and limit of detection comparable to ELISA^19^. We compared sensitivity of detecting anti-p53 autoantibodies using NAPPA on microreactor array vs. flat glass. Full length genes for p53 protein were printed on both microreactor array and flat glass and processed as described in **Methods**. Printing conditions were optimized for their respective substrates, i.e. piezoelectric dispensing for microreactor arrays and pin-spotting for flat glass. The two types of substrates were then probed with serum sample from the same colon cancer patient at four serum dilutions from 1:50 to 1:900. Signal to background ratio of anti-P53 response is five times higher for NAPPA on microreactor array vs. standard flat glass, Figure 5. Signal to background ratios in Figure 5 are much lower than Figure 4 since serum has many different proteins that bind nonspecifically to the surface and increase background.

**Figure 6:**
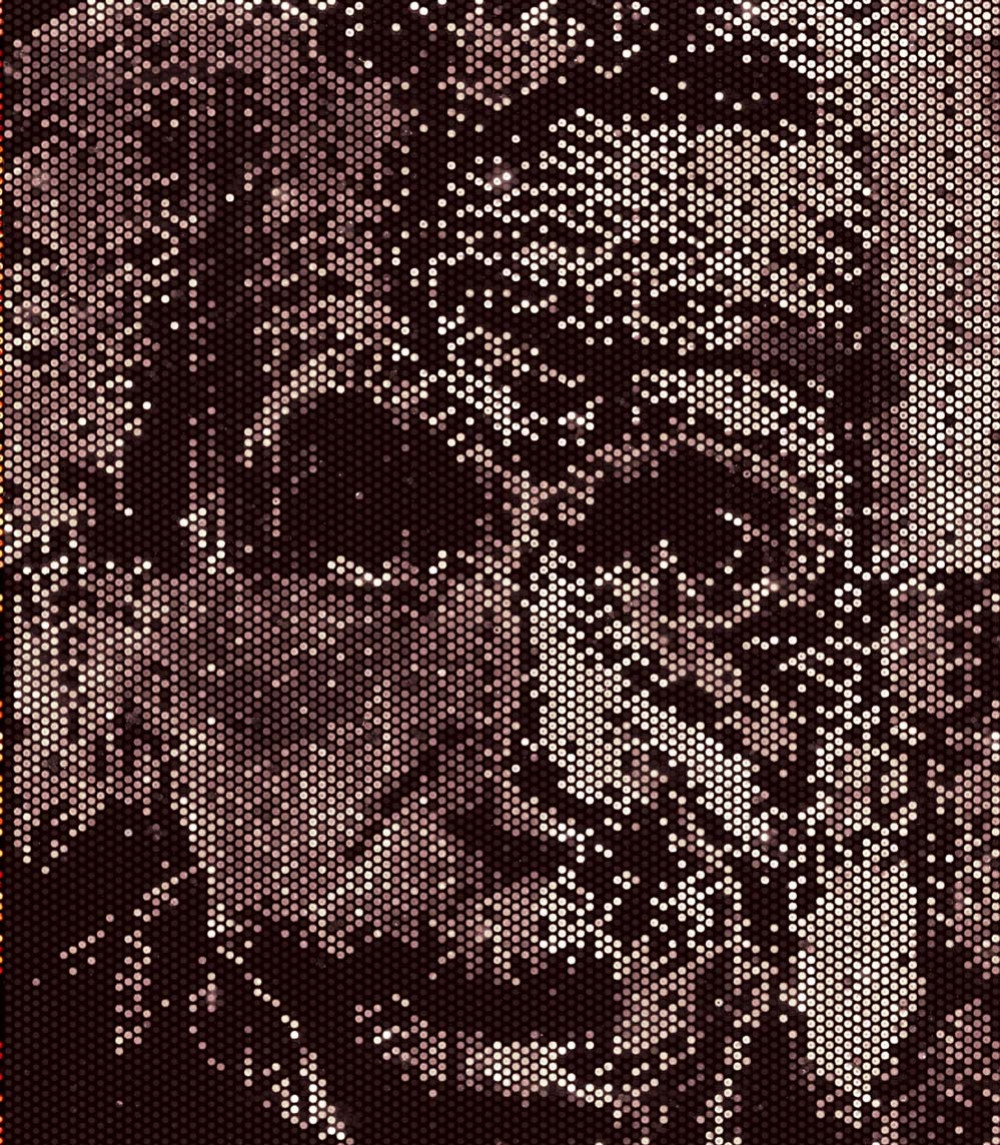
Image rendered with proteins expressed from DNA in microreactors. Four concentrations of plasmid DNA for the p53 gene are printed into small chemical reaction chambers (microreactors) on a microscope slide format using a PiXY piezoelectric dispensing instrument. Proteins are expressed *in situ* from the DNA using *in vitro* coupled transcription and translation (IVTT) reagent. Microreactors are sealed during three hours protein expression to prevent diffusion. Varying concentrations of DNA produce varying signal intensities of fluorescently labeled protein spots. Black spots are empty. The image has ~12 kilopixel resolution (108 × 108 spots). It was initially split in half to print on a microscope slide format. Original photograph of Albert Einstein by Philippe Halsman (c) Halsman Archive.

A bitmap image rendered in fluorescently labeled p53 protein expressed in microreactors from plasmid DNA for the p53 gene is shown in Figures 6. The image illustrate fidelity of the microreactor array platform with density greater than 10,000 protein spots expressed on a single microscope slide format with no diffusion or evaporation during three hours incubation. Moreover, the ability to control different protein levels by adjusting the amount of printed DNA, demonstrates the nuanced ability of these microreactors to test reactions under quantitative conditions.

## Discussion

### Previous work

The microreactor array is a synergy of microplate and microarray technology. It miniaturizes microplates^1^ down to the scale of microarrays^20^,^21^,^22^,^23^,^24^ allowing tens of thousands independent biochemical reactions simultaneously on a single microscope slide format. It is based on an array of microreactors that are accessed in parallel by a thin sheet of reagent and then sealed by viscous liquid acting through a flexible sealing membrane. To our knowledge, this micofluidic configuration is the first of its kind. It does not follow the conventional ‘lab-on-a-chip’ paradigm, i.e. a miniaturized, serial, 2D network of micro-channels emulating an electronic circuit or conventional chemical processing plant. Such platforms are also known as ‘micro total analysis systems’ (μTAS), ‘micro electrical mechanical systems’ (MEMS) or ‘microreactors’^25^,^26^,^27^,^28^,^29^. They were originally developed for polymerase chain reaction (PCR)^30^,^31^. Various planar microreactor array type platforms have also been developed for PCR^32^,^33^,^34^,^35^,^36^,^37^,^38^,^39^,^40^,^41^,^42^,^43^ and other life science applications^44^,^45^,^46^,^47^,^48^,^49^,^50^,^51^,^52^ but none use the unique microfluidic configuration presented here. Cell-free *in vitro* protein expression has been done in various microreactor formats^53^,^54^,^55^,^56^,^57^,^58^,^59^ but never using the present configuration for filling and sealing the microreactors.

### Microreactor filling

Physical design principles to effectively fill the array of microreactors with reagent are presented based on fluid mechanics nondimensional analysis. Reagents in the life sciences are typically aqueous solutions. Water has high surface tension which dominates other forces at the small dimensions of microreactors making them difficult to fill with reagent. For the microreactor array platform, reagent is forced into microreactors by first applying vacuum to a thin fluid gap between the microreactor array slide surface and sealing membrane and then quickly injecting reagent, under pressure, into this gap. This approach fills microreactors by overcoming surface tension through a combination of vacuum, pressure, inertial, wetting and viscous forces. Kinetic and potential energy of the reagent overcomes its surface energy. Similar issues, concerning filling small features with fluids, arise in other fields such as plastic injection molding and microimprint lithography^60^.

Weber number 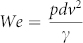 characterizes relative influence of inertia vs. surface tension of fluid with density *ρ* and surface tension *γ* flowing with velocity *v* in a channel with hydraulic diameter *d*. Weber number is used to analyze thin film flows and the formation of droplets and bubbles^61^. The Weber number for the microreactor array platform is *W_e_* ≌ 0.02 meaning that inertial forces have low influence on filling microreactors compared to surface tension.

Capillary number 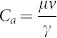 characterizes relative influence of viscous vs. surface tension forces. The microreactor array platform has low capillary number *C_a_* ≌ 0.001 meaning that reagent flow dynamics at velocity *v* are heavily influenced by surface tension *γ* compared to dynamic viscosity μ. The following key conditions are identified for complete filling of small features^62^: high Capillary number, low aspect ratio features and high surface wetting of the features vs. the opposing surface. Aspect ratio is relative depth to width ratio of microreactors. Generally, aspect ratios less than one are required for complete filling of microreactors^62^. A higher wetting (hydrophilic) microreactor array slide surface compared to the opposing surface (hydrophobic) promotes filling of the microreactors^63^.

Reynolds number 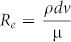 characterizes relative influence of inertial vs. viscous forces. Physical design parameters are interdependent and must be balanced for good filling of the microreactors. High reagent injection velocity increases Capillary number to help overcome surface tension with viscous forces. However Weber number also increases with velocity resulting in incomplete filling of microreactors due to inertial forces which can break up reagent flow^62^. This manifests itself as foaming and is exacerbated by gas dissolved in the reagent. Inertia can also cause reagent to skim across the top of the microreactors instead of flowing down into them. Complete filling of microreactors is therefore assisted by relatively low Weber number and relatively high Capillary number. The ratio of Weber to Capillary numbers also corresponds to Reynolds number 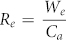 which should therefore be low for complete filling of microreactors. Regent flow in the fluid gap of the microreactor array platform has relatively low Reynolds number *R_e_* ≌ 20 resulting in laminar flow. Low Reynolds number corresponds to low reagent injection velocity *v* high kinematic viscosity 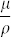 and small reagent fluid gap 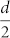.

Physical parameter values for water are used here to calculate the non-dimensional numbers. Filling microreactors with actual reagents may be assisted by thickening agents to increase viscosity (lower *R_e_*) or surfactants to decrease surface tension (higher *C_a_*). Small concentrations of surfactants can dramatically decrease surface tension.

### Microreactor sealing

Microreactors are sealed with viscous liquid acting through a flexible sealing membrane, Figure 1. Viscous liquid has the appropriate combination of physical properties to seal the microreactors: incompressible flow to displace excess reagent from the microreactor array surface followed by isotropic pressure to seal all of the microreactors uniformly. This is the first time that viscous liquid is used in this manner and is a key advancement for this process. It provides a simple elegant solution to a challenging problem.

As shown in Figures 3 & 4, alternate methods for sealing microreactors are not effective in preventing diffusion between microreactors. Using silicone rubber is the best alternate method but it is not completely effective. Silicone rubber presses down uniformly on the microreactor surface invariably entrapping reagent in small pockets that prevent complete sealing of the microreactors in those areas. Using either gas or hydraulic oil is not effective since each one quickly spreads out across the sealing membrane entrapping a layer of reagent that again prevents complete sealing of the microreactors. Compared to the preferred method of using viscous sealing liquid, the alternate methods clearly demonstrate the need for an effective means of first wiping reagent away from the surface before sealing the microreactors.

### Conclusion

For NAPPA, the microreactor array platform provides an array of miniaturized reaction chambers that can be individually programmed to produce any desired protein. NAPPA is commonly used to screen antibody affinity to those proteins in serum samples from healthy vs. diseased individuals. The microreactor array platform has higher signal to background ratio of antibody response compared to standard NAPPA on flat microscope slides, Figure 5. It can therefore detect serum antibodies with higher sensitivity and potentially improve the accuracy of clinical studies correlating antibody response to disease.

## Methods

Methods to generate NAPPA protein microarrays using microreactor arrays with the preferred fill & seal device of Figure 1 are presented. Methods to screen for antibodies in patient serum samples and analyze the results are also presented.

### Fabricate microreactor arrays

#### Take appropriate safety precautions

To fabricate microreactor array slides, work in a properly equipped semiconductor fabrication cleanroom (Center for Solid State Electronics Research (CSSER), Arizona State University, Tempe AZ). For biochemistry procedures, work in a biosafety level 1 (BSL-1) laboratory (Center for Personal Diagnostics (CPD), Biodesign Institute, Arizona State University, Tempe AZ). Work in a BSL-2 lab (CPD) for serum screening assays. Acquire appropriate training and certification for the facilities, procedures, equipment, chemicals, samples and wastes. Obtain all chemicals from Sigma Aldrich (St. Luis MO) except where noted.

#### Design photolithography mask for microreactor array slides

Generate a drawing for the photolithography mask using computer aided design (CAD) software (AutoCad, Autodesk, San Rafael CA). Draw a 150 mm diameter circle. Space seven microscope slide format slides 25.4 mm × 76.2 mm apart within the circular outline. Add 50 μm wide cut-lines ‘streets’ between slides for dicing. For each slide, draw ~14,000 130 μm diameter circles for the microreactors in a hexagonal closest packing array pattern with 375 μm center-to-center spacing. Leave at least 1.33 mm empty areas, without features, at the top and bottom of each slide. These areas are used for autofocus by the microarray scanner. Put numbers along the sides and bottom of each slide to identify rows and columns respectively. Include a logo along the bottom of each slide to orient it during the various processing steps of printing, assaying and scanning. Add 200 μm diameter circles outside of the slides for depth measurement during the etching process.

#### Fabricate photolithography mask for microreactor array slides

Upload photolithography mask drawing to mask manufacturer (JD Photo-Tools, Oldham UK). Specify ‘7″ × 7″ chrome glass’, ‘super-high resolution’ (128 K dpi), ‘darkfield’ and ‘design viewed from glass side’. Fabricate the mask. Clear areas of the mask (no chrome) correspond to the etched areas of the silicon wafer in subsequent steps.

#### Pattern silicon wafers

Obtain 6″ (150 mm) silicon wafers (University Wafer, Boston MA) with standard thickness of 675 μm +/− 25 μm. Electrical properties and crystal orientation of the silicon do not matter. Coat one side of each wafer with 300 nm LPCVD low stress nitride. Spin coat nitride with 1 μm AZ 3312 (AZ Electronic Materials Inc., Branchburg NJ) positive photoresist. Bake at 100°C for two minutes. Expose photo resist with photolithography mask on mask aligner (OAI, San Jose CA). Develop photoresist in AZ300 MIF developer (AZ Electronic Materials Inc., Branchburg NJ) for 45 seconds and hard-bake at 100°C for 2 minutes. Selectively etch nitride film with reactive ion etch (RIE). Remove photoresist with acetone.

#### Etch microreactors

Prepare HNA etchant mixture of 49% hydrofluoric acid (HF), 70% nitric acid (HNO_3_) and (>98%) glacial acetic acid (CH_3_COOH) in the ratio of 2.75:1.75:1. Isotropically etch wafers for ~30 minutes to 70 μm depth and 270 μm diameter. Etching silicon with HNA is exothermic so agitate the wafers to maintain uniform temperature.

#### Grow oxide layer

Clean surface in piranha mixture (1:1 mixture sulfuric acid (H_2_SO_4_): hydrogen peroxide (H_2_O_2_)) for 15 minutes. Clean surface in buffered oxide etch of hydrofluoric acid (HF) and ammonium fluoride (NH_4_F) (1:6 mixture of HF:NH_4_F) for 10 seconds. Grow 95 nm thin film silicon dioxide (SiO_2_) layer at 1,000°C for ~1 hour in oxygen furnace (Tystar 4600, Torrance CA).

#### Dice wafers

Dice wafers along cut-lines (Advotech Company, Inc., Tempe AZ) into 7 individual microreactor array slides. Saw blade kerf is 50 μm so actual slide dimensions are 25.35 mm × 76.15 mm.

#### Functionalize surfaces

Clean residual organic materials from the microreactor array slides in piranha (1:1 mixture of H_2_S0_4_:H_2_O_2_) for 30 minutes. After rinsing with DI water and drying with compressed air, immerse slides in 2% solution of (3-Aminopropyl) triethoxysilane (APTES) in acetone for 30 minutes. Thoroughly rinse slides in acetone and then DI water. Dry the slides with compressed air.

### Print DNA into microreactors

#### Obtain plasmid DNA

Obtain a set of genes to be printed from DNASU Plasmid Repository (Center for Personal Diagnostics, Biodesign Institute, Arizona State University, Tempe AZ). Clones are sequence verified and inserted in the E.Coli pDNR-dual recombinational cloning vector open reading frames (ORFs) with the natural stop codon absent and GST-tag appended to C-terminus.

#### Purify plasmid DNA

Grow E.Coli colonies of clone vectors. Harvest E.Coli and purify plasmid DNA in 96 microreactor miniprep (Whatman Filter Plates, Sigma-Aldrich, St. Louis MO). Transfer miniprep DNA to 384-well microplate.

#### Normalize plasmid DNA

Normalize miniprep plasmid DNA to 100 ng/μl (Nanodrop 8000, Thermo Scientific, Wilmington DE).

#### Store plasmid DNA

Store plasmid DNA at 4°C until printing.

#### Prepare NAPPA printing-mixture

Prepare 1X printing-mixture. Thaw components and keep them on ice. Mix components together in the following order and proportions: 93% nuclease-free DEPC-treated water (Ambion, Life Technologies, Grand Island NY), 0.6% BSA (Sigma-Aldrich), 1% anti-GST antibody (GE Healthcare), 5% BS3 crosslinker (Thermo Scientific, Pierce). Keep mixture on ice. Age printing-mixture at 4°C for one day to allow partial crosslinking of BS3. Aliquot printing-mixture into 384-well microplate for printing. Keep microplate on ice until ready to print.

#### Prepare microarray pattern

Predefine a microarray printing pattern for the 96 plasmid DNA and the printing-mixture. Surround each spot containing printing-mixture and plasmid DNA with 6 spots containing just the printing-mixture without the plasmids. In this way, expressed proteins that diffuse away from a plasmid spot are captured by the anti-GST antibodies in the printing-mixture of neighboring spots and then easily detected.

#### Prepare bitmap pattern

For Figure 6 resize digital RGB image to 108 × 108 pixels using function imresize() (Matlab, MathWorks, Natick MA). Using imrgb2ind() reduce image to 5 uniformly spaced grayscale intensities, gray(5). Split image into two halves to fit on a 1″ × 3″ microscope slide format. Convert to bitmap file using imind2rgb().

#### Align microreactor array slides

Align APTES functionalized microreactor array slides on the deck of a non-contact piezoelectric dispensing microarrayer (Rainmaker-au302, Engineering Arts LLC, Tempe AZ). Use the au302 alignment system to align the microreactors of the slides for continuous non-contact dispensing.

#### Print microreactor array slides

Use either 8 or 16 piezoelectric dispensers (Engineering Arts LLC, Tempe AZ) on the au302 microarrayer. Prime dispensers with DI water. Place the 384-well microplate with printing-mixture on the deck of the au302 microarrayer. Aspirate 2 μl printing-mixture into each dispenser using on-head aspiration syringes. Dispense twelve 0.1 nl drops of printing-mixture in short bursts at 12,500 drops-per-second into microreactors using the predefined microarray pattern defined above. Use non-contact piezoelectric on-the-fly dispensing at uniform print-head speed of 175 mm/sec. Dispense the same number of drops with the same predefined pattern on flat microscope slides for Figure 2B. Clean the piezoelectric tips by flushing DI water through the dispenser while the dispenser tip is submerged in flowing DI water. Within 10 minutes, repeat the same process with the 384-well microplate of plasmid DNA. Print 3 drops of plasmid DNA in the same spots as the printing-mixture. Use the same aspirating and dispensing parameters (12,500 drops-per-second) as for the printing-mixture. Do not print plasmid DNA into the 6 surrounding microreactors containing just printing-mixture alone. Repeat the process of dispensing printing-mixture followed by plasmid DNA until all of 96 genes are printed. DNASU plate 4156 containing 96 general purpose genes was printed on the slide in Figure 2A. Print on flat glass at the same time for Figure 2B. Print plasmid DNA for the p53 gene onto the slide in Figure 6 using a PiXY piezoelectric microarrayer that prints colored bitmaps.

#### Print flat glass slides for p53 serum screening comparison

Mix DNA and printing mixture together. Print mixture on APTES coated glass microscope slides (Fischer Scientific, Waltham MA, Cat. No. 12-544-1) using Genetix QArray2 pin-spotter at 2,500 spot density per microscope slide.

#### Store microarrays

Store printed plasmid DNA microarrays at room temperature in a sealed container with desiccant. Printed plasmid DNA microarrays can be stored for several months before protein expression.

### Express proteins

#### Block microreactor array slides

This step also washes away unbound printed molecules from slides' surfaces. Program centrifuge (Beckman Coulter model Allegra X-15R, Indianapolis IN) for 3750 RPM, maximum acceleration and maximum deceleration. Submerge slides in a tray containing 8 ml of Superblock Blocking Buffer in TBS (Thermo Scientific product #37535B, Rockford IL). Accelerate up to the maximum speed and decelerate right away. Rinse slides in DI water and dry them with compressed air.

#### Block glass slides

Use the same procedure as outlined above for microreactor array slides. However, the centrifuge is not required since glass slide surfaces are flat so they do not entrap air. Block slides for one hour on a rocking table (VWR model 200, Radnor PA).

#### Prepare *in vitro* coupled transcription and translation (IVTT) reagent

Prepare IVTT reagent from the “1-step human *in vitro* protein expression kit” (Thermo Scientific, Rockford, IL). Thaw out the four components of the kit and keep them on ice. Mix components in the following order and proportions: 34% HeLa lysate, 10% accessory proteins, 22% reaction mix and 34% nuclease-free water. This uses 60% HeLa lysate compared to the normal recipe. Keep mixture on ice. Degas lysate in a vacuum desiccator until bubbles are gone in approximately 5 minutes.

#### Apply IVTT reagent to microreactor array slides

Insert a microreactor array slide into the fill & seal device of Figure 1 (Biodesign Institute, ASU, Tempe AZ). Put the O-ring around the slide. Place sealing membrane on top of the O-ring and slide. Place window on top of the sealing membrane and clamp the fill & seal device frame together using fasteners. Apply vacuum to the reagent outlet port and inject 300 μl IVTT reagent into the inlet/outlet port. Inject pressurized sealing liquid into the inlet port in the window to displace the reagent and seal the microreactors.

#### Apply IVTT reagent to flat glass slides

Attach gasket (Hybriwell Sealing System item 440904, Grace Biolabs, Bend OR) to the slide. Using manual pipette, inject 150 μl IVTT reagent into the hole at one end of the gasket.

#### Incubate glass slides

Incubate glass slides and gasket at 30°C in a chilling incubator (EchoTherm chilling incubator, Torrey Pines Scientific, Carlsbad CA) for 1.5 hours and then 15°C for 0.5 hours.

#### Incubate microreactor array slide

Incubate the fill & seal device assembly at 30°C in a chilling incubator (EchoTherm chilling incubator, Torrey Pines Scientific, Carlsbad CA) for 2 hours and then 15°C for 1 hour. Incubation times are longer than standard NAPPA on glass slides to accommodate longer heating and cooling times due to the higher thermal mass of the fill & seal device.

### Detect fluorescent signals

#### Fluorescently label proteins

To preserve molecular functionality, do not allow slides to dry out between processing steps. Prepare 5% milk-PBST (0.2% Tween) blocking buffer. Combine 500 ml 1x phosphate buffered saline (1 × PBS) with 25 grams 100% instant nonfat powdered dry milk and 1 ml Tween 20 detergent and mix with magnetic stirring bar for 10 minutes. Store at 4°C. Fill a small tray with blocking buffer. Disassemble the fill & seal device, remove the slide and submerge it into blocking buffer. Block for one hour at room temperature on a rocking table (VWR model 200, Radnor PA) replacing blocking buffer three times. For Figures 3 & 4, prepare primary label: mix 10 μl mouse anti-GST monoclonal antibody (Cell Signaling Technologies, Danvers MA) in 3 ml blocking buffer. Incubate slides in primary label overnight at 4°C on a rocking table. Rinse slides with blocking buffer 3 times for 10 minutes each time; replacing blocking buffer each time. Prepare secondary label: mix 6 μl Alexa Fluor 647 nm goat anti-mouse IgG antibody (Life Technologies, Grand Island NY) in 3 ml blocking buffer. Incubate slides in secondary label for 1 hour at room temperature on a rocking table in the dark to prevent photo bleaching of the fluorescent dye. Prepare rinsing buffer: 500 ml 1 × PBS and 1 ml Tween. Rinse slides in rinsing buffer three times for 1 minute each time; replacing the rinsing buffer each time. Rapidly rinse slides in DI water 6 times replacing water each time. Dry slides with compressed air. Store slides in the dark, at room temperature with a desiccator.

#### Probe serum samples

Following protein expression, block slides in 5% milk-PBST (0.2% Tween) on a rocking table at room temperature for 1 hour. For Figure 5, incubate slides with diluted serum sample in proplate 4-well tray set (Grace Bio-Labs, OR) at 4°C overnight. Wash slides in 5% milk-PBST (0.2% Tween) three times, 5 min each time. Detect protein display with Alexa Fluor 647 labeled goat anti-human IgG secondary antibody (Jackson ImmunoResearch, West Grove, PA). Finally, wash slides in DI water and dry them with compressed air.

#### Image microreactor array slides

Program fluorescent microarray scanner (PowerScanner, Tecan, Mannedorf Switzerland) for microreactor array slides. Define coordinates of the empty areas at the top and bottom of slides for autofocus. Define reflectivity of those areas. Define 70 μm offset for microreactor depth after autofocus. Apply self-adhesive 325 μm thick backing to slides to accommodate 1 mm slide thickness requirement of the scanner. Scan slides at 10 μm resolution, 25% laser power (out of 30 mW) and 25% (out of 800% maximum) photo multiplier tube (PMT) detector gain. Extract data (Array-Pro, Media Cybernetics, Rockville MD). Analyze data (Excel, Microsoft, Redmond WA). Plot signal to background ratio of anti-P53 response, Figure 5. Signal for each dilution point is the average signal of six spots on the array. Signal of each spot is the median pixel value of that spot. Background is the median signal of all of the spots on the array.

## Author Contributions

P.W. developed the preferred and alternate fill & seal devices, rendered Figures 1, 2, 3, 4, 6, printed the microarray for Figure 6, ran validation assays on the fill & seal devices and wrote the main text. A.B. provided technical insight, printed microarrays for Figure 3 and ran validation assays. P.K. printed microarrays for Figure 3. J.Q. provided DNA preps, serum samples and directed serum screening. M.M. provided technical direction for NAPPA chemistry. X.B. screened serum samples, provided data analysis and rendered Figure 5. K.K. ran validation assays and provided data analysis. J.L. provided technical direction for NAPPA. All authors reviewed the manuscript.
